# Response to “Letter to the Editor Regarding Prognostic Impact of Hepatectomy Versus Radiofrequency Ablation for Non‐Small Hepatocellular Carcinoma (2–3 cm): A Case‐Matched Study”

**DOI:** 10.1002/ags3.70126

**Published:** 2025-11-14

**Authors:** Yuki Kitano, Hiromitsu Hayashi, Takumi Tanizaki, Yoshiyuki Tagayasu, Takashi Matsumoto, Rumi Itoyama, Shigeki Nakagawa, Hirohisa Okabe, Masaaki Iwatsuki

**Affiliations:** ^1^ Department of Gastroenterological Surgery, Graduate School of Medical Sciences Kumamoto University Kumamoto Japan

**Keywords:** alpha fetoprotein, hepatectomy, hepatocellular carcinoma, radiofrequency ablation

We sincerely appreciate the thoughtful comments of Dr. Chen and colleagues regarding our recent article entitled “Prognostic Impact of Hepatectomy Versus Radiofrequency Ablation for Non‐small Hepatocellular Carcinoma (2–3 cm): A Case‐Matched Study” in relation to our research group's original paper published in the *Annals of Gastroenterological Surgery* [1]. We are grateful for their interest in our work and for highlighting the important clinical considerations in the treatment of hepatocellular carcinoma (HCC).

The choice between hepatectomy and radiofrequency ablation (RFA) is influenced by liver functional reserve and tumor location. Poor liver function often necessitates RFA, whereas tumors on the liver surface or adjacent to neighboring organs or vessels are frequently managed by surgical resection. We concur that the proximity to major vessels can compromise RFA efficacy owing to the well‐known heat sink effect, as demonstrated by Lee et al. [2]. In line with previous reports, our institutional policy has been to avoid RFA for tumors adjacent to vascular structures because of its reduced therapeutic effect. This approach likely minimizes the influence of perivascular tumors on our outcomes and reflects common clinical practice. Furthermore, we provided additional details regarding surgical margins. In our hepatectomy cohort, the mean surgical margin was 8.5 ± 1.0 mm. While this margin is narrower than the “wide margin” (approximately 2 cm) shown to improve outcomes in a randomized trial by Shi et al. [3], it remains wider than the 5‐mm ablation margin in our RFA cohort. This intermediate margin may partially explain the superior local tumor control observed after hepatectomy. Although our margins were closer to the “narrow margin” group, they still showed oncologic benefit over RFA, supporting hepatectomy as the preferred option whenever technically and functionally feasible. Moreover, because our study population, after propensity score matching, included a relatively high proportion of patients with impaired liver function, the margins in the hepatectomy cohort were inevitably narrower than those achievable in patients with better‐preserved function.

Our study primarily aimed to evaluate patients with tumors 2–3 cm in size, as more than 60% of patients in the SURF trial had tumors < 2 cm [4]. Even after adjustment by propensity score matching, hepatectomy yielded significantly lower local recurrence rates and a survival benefit in patients with elevated alpha‐fetoprotein (AFP ≥ 20 ng/mL). These findings indicate that biological and technical factors, including AFP levels, tumor location, and achievable resection margins, should guide individualized treatment decisions.

We acknowledge that our retrospective, single‐institution study design has inherent limitations. Larger multicenter prospective studies are warranted to validate these findings and clarify the optimal margin thresholds in different clinical scenarios. Furthermore, although our institutional policy is to secure an ablation margin of at least 5 mm for RFA whenever possible, this retrospective design did not allow accurate measurement. Post‐RFA CT was not performed to all cases, and even when available, the absence of a visible target lesion made it difficult to determine the exact margin. Nonetheless, our data add to the existing evidence that hepatectomy provides superior local control compared with RFA, even when the margins are modest, and should be considered the preferred option for patients with 2–3 cm HCC when feasible.

Again, we sincerely thank Dr. Chen et al. for their constructive comments. We hope that our findings, together with their insightful comments, will contribute to more evidence‐based and individualized strategies for HCC management.

## Author Contributions

**Yuki Kitano:** conceptualization, investigation, writing – original draft, methodology. **Hiromitsu Hayashi:** conceptualization, writing – review and editing. **Takumi Tanizaki:** writing – review and editing. **Yoshiyuki Tagayasu:** writing – review and editing. **Takashi Matsumoto:** writing – review and editing. **Rumi Itoyama:** writing – review and editing. **Shigeki Nakagawa:** writing – review and editing. **Hirohisa Okabe:** writing – review and editing. **Masaaki Iwatsuki:** conceptualization, writing – review and editing.

## Ethics Statement

The study protocol was approved by the Ethics Committee of Kumamoto University (IRB No. 1291).

## Consent

All patients provided written informed consent.

## Conflicts of Interest

The authors declare no conflicts of interest.

## Linked Articles

This article is linked to Changzhi et al. papers. To view this articles, visit https://doi.org/10.1002/ags3.70075.
